# Nickel-Doped Cerium Oxide Nanoparticles: Green Synthesis Using *Stevia* and Protective Effect against Harmful Ultraviolet Rays

**DOI:** 10.3390/molecules24244424

**Published:** 2019-12-04

**Authors:** Mehrdad Khatami, Mina Sarani, Faride Mosazadeh, Mohammadreza Rajabalipour, Alireza Izadi, Meghdad Abdollahpour-Alitappeh, Marcos Augusto Lima Nobre, Fariba Borhani

**Affiliations:** 1 NanoBioEletrochemistry Research Center, Bam University of Medical Sciences, Bam 76617-71967, Iran; mehrdad7khatami@gmail.com (M.K.); minasarani64@gmail.com (M.S.); mmrr1366@yahoo.com (M.R.); 2 Cell Therapy and Regenerative Medicine Comprehensive Center, Kerman University of Medical Sciences, Kerman 7618747653, Iran; 3 Students Research Committee, School of Public Health, Bam University of Medical Sciences, Bam 76617-71967, Iran; f.mousazadeh7@gmail.com; 4 Department of Medical Parasitology and Mycology, School of Public Health, Tehran University of Medical Sciences, Tehran 14167-53955, Iran; alirezaizadi81@yahoo.com; 5 Cellular and Molecular Biology Research Center, Larestan University of Medical Sciences, Larestan 74319-75566, Iran; abdollahpour1983@yahoo.com; 6 School of Technology and Sciences, São Paulo State University (Unesp), Presidente Prudente-SP 19060-900, Brazil; marcos.nobre@unesp.br; 7 Medical Ethics and Law Research Center, Shahid Beheshti University of Medical Sciences, Tehran 19857-17443, Iran

**Keywords:** Ni-doped CeO_2_ nanoparticles, *Stevia rebaudiana*, UV protection, VSM

## Abstract

Nanoparticles of cerium oxide CeO_2_ are important nanomaterials with remarkable properties for use in both industrial and non-industrial fields. In a general way, doping of oxide nanometric with transition metals improves the properties of nanoparticles. In this study, nickel- doped cerium oxide nanoparticles were synthesized from *Stevia rebaudiana* extract. Both doped and non-doped nanoparticles were characterized by X-ray diffraction, Field Emission Scanning Electron Microscopy, Energy Dispersive X-ray, Raman spectroscopy, and Vibrating-Sample Magnetometry analysis. According to X-ray diffraction, Raman and Energy Dispersive X-ray crystalline and single phase of CeO_2_ and Ni doped CeO_2_ nanoparticles exhibiting fluorite structure with F2g mode were synthesized. Field Emission Scanning Electron Microscopy shows that CeO_2_ and Ni doped nanoparticles have spherical shape and sizes ranging of 8 to 10 nm. Ni doping of CeO_2_ results in an increasing of magnetic properties. The enhancement of ultraviolet protector character via Ni doping of CeO_2_ is also discussed.

## 1. Introduction

Nowadays, research and development of products in the nanotechnology field has been steadily increased, in specific due to innovative and beneficial properties of materials at the nanoscale. Furthermore, nanotechnology as a flexible tool can be applied in electrical devices, construction of composite materials, catalysts and antibacterial coatings [1]. Nanoparticles constitute the fundamental structures of nanotechnology [2]. A most relevant feature of nanoparticles stems from their large ratio surface-to-volume ratios [2]. Nanoparticles provide a higher surface area-to-volume ratio through reduction of their particle size, which increases their efficacy in biological media. Among oxide type nanomaterials, cerium oxide nanoparticles can be utilized in sensors, catalysts, sunscreens, ultraviolet physical absorbers and sun cells [3,4], because of their high thermal stability, wide band-gap and rapid changing of oxidation states between Ce (III) and Ce (IV) [3].

The human skin acts as an effective barrier against the harmful effects of environmental substances [5]. In addition, exposure to ultraviolet radiation is a key factor to human skin problems such as cracks, burns, immunosuppression, wrinkles, dermatitis, inflammation, aging, hypopigmentation, hyperpigmentation and skin cancers [6]. Sunscreens are one of the most common options for sunlight protection because of their ease of use and higher effectiveness in radiation protection [7]. Mineral sunscreens contain inorganic UV filters, ultraviolet absorbers, like titanium dioxide TiO_2_ and zinc oxide ZnO, which have been safe and effective [8]. Oxides of the zinc oxide, cerium oxide; titanium dioxide and iron oxide type are UV light absorbers. Some previous studies involving nanoparticles for UV protection have shown that the absorber efficiency of the oxide depends in a major part on the particle size [9]. In addition to the conventional TiO_2_ and ZnO filters, the development of new filters has also been carried out to find new consumer products. The next-generation sunscreen filters with advanced features should be more efficient and safer for the health of users and the environment, replacing TiO_2_ and/or ZnO. At the moment seems that cerium oxide nanoparticles are a promising mineral for use as a UV filter in sunscreen cosmetics [10]. Several methods of chemical synthesis such as sol-gel, precipitation, thermal decomposition have been employed to attain cerium oxide nanoparticles [11,12,13,14]. However, a major part of these synthesis methods are not environmentally friendly, making green chemistry methods like the biosynthesis of nanoparticles using plants and plant products fundamental as a function of benefits such as low cost, safe and environmental security [15,16,17,18,19,20,21,22,23,24,25]. In this sense, *Stevia rebaudiana* (Asteraceae) is a promising plant to support biosynthesis. It is a shrub with a height of one meter. The plant has small leaves that alternate on the stem [26]. The leaves of this plant have diterpene glucosides, which are naturally sweet. Its sweet components are stevioside, steviol bioside, ribodiazide and dalcoside [27]. Recent investigations have shown that this plant has the ability to prepare in a fast and non-toxic way chemically stable iron oxide, zinc oxide and zinc sulfide nanoparticles [28,29,30,31,32,33].

In this study, a biosynthesis method based on aqueous extract of *Stevia* to prepare functional nanoparticles was implemented. Syntheses of CeO_2_ oxide and of Ni-doped CeO_3_ solid solutions were carried out. UV protection of the resulting products was measures as Sun Protection Factor (SPF).

## 2. Results and Discussion

**Figure 1:**
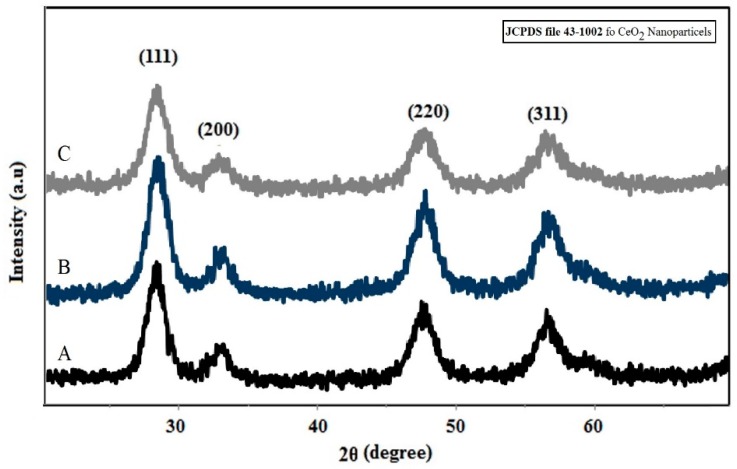
X-Ray diffraction profile of synthesized nanoparticles of (**A**) CeO_2_ and CeO_2_ doped with 1% Ni (**B**) and 3% Ni (**C**) by using *S. rebaudiana* extract as synthesis medium.

The X-ray diffraction curves of CeO_2_ and Ni-doped CeO_2_ powder are shown in Figure 1. According to JCPDS file 43-1002, all detected diffraction lines correlated to the following Müller indices (111), (200), (220) and (311), ascribed to the fluorite cubic structure. A specific broadening of diffraction lines presents at all diffraction lines indexed was attributed to the nanometric scale of the particles. A slight shift in the angle position of the (111) diffraction line was identified for all diffraction lines of the CeO_2_ structure doped with Ni. The crystallite size was estimated using Scherrer equation D = 0.89λ/βcosθ; where D is the crystallite size of a particle, λ is X-ray wavelength, β is full width at half maximum height and θ is the angle of diffraction. All synthesized powders are nanometric in size, being the CeO_2_ average crystallite size equal to 10.56 nm. The crystallite size as a function of Ni doping level show that crystallite size decreased with increasing of Ni concentration in the cubic structure of CeO_2_.

**Figure 2:**
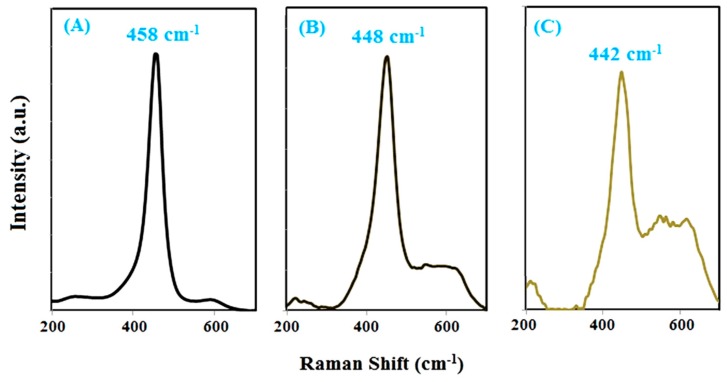
Raman spectra synthesized nanoparticles of (**A**) CeO_2_ and CeO_2_ doped with 1% Ni (**B**) and 3% Ni (**C**) by using *S. rebaudiana* extract.

Raman spectroscopy is a powerful tool for characterizing molecular fingerprints and monitoring changes in molecular bond characteristics and their effects on the crystalline structure e.g., stresses and/or strains, crystalline form and crystallinity. CeO_2_ has a single active mode type F2g that is usually positioned at around 463.08 cm^−1^ [34]. This vibrational mode is related to the structure of fluorite, and any type of changes in the ions in this structure changes the intensity and vibrational position of this mode. The Raman spectrum of cerium oxide nanoparticles (Figure 2) shows the F2g mode is positioned at 458 cm^−1^, which indicates the fluorite structure for these nanoparticles, as discussed in the XRD analysis section. The development of solid solutions via Ce substitution by nickel ions into fluorite structure of CeO_2_ changed the position and intensity of this mode.

With the increase of doped nickel content, the mode position shifted to lowest values, while its intensity decreased in a substantial way [35]. These find are in accordance with the discussion in the X-ray diffraction section that showed a decrease of crystallinity accompanied of an increase of microstrain level and a decrease in crystallite size.

**Figure 3:**
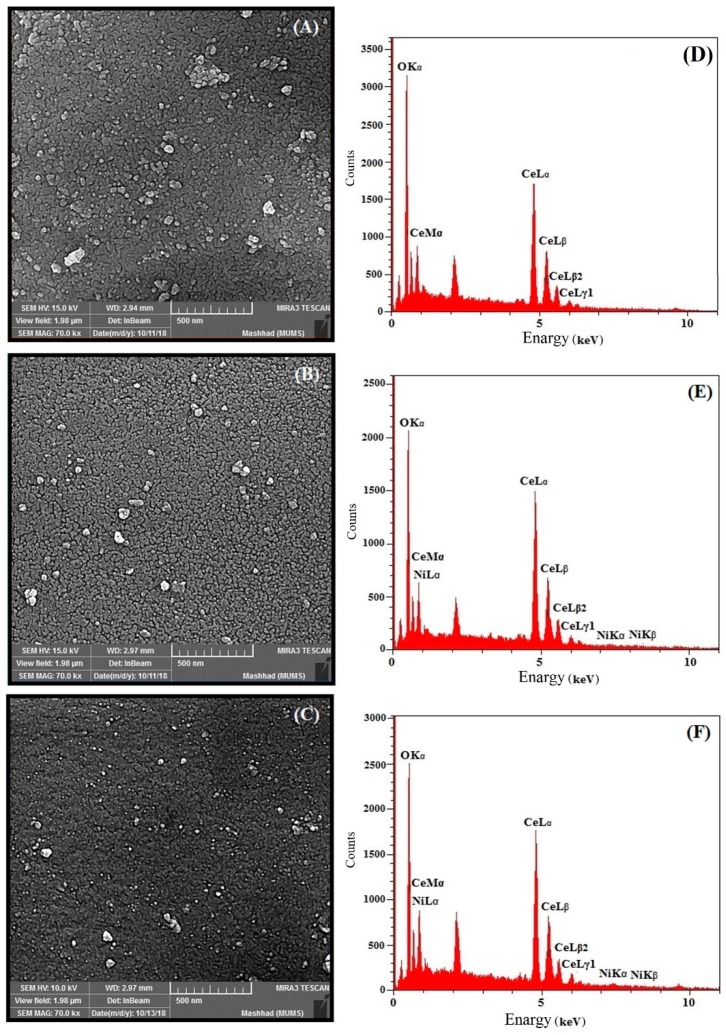
FESEM images of synthesized nanoparticle of (**A**) CeO_2_ and CeO_2_ doped with 1% Ni (**B**), 3% Ni and (**C**); and EDX graph of synthesized nanoparticles of (**D**) CeO_2_ and CeO_2_ doped with 1% Ni (**E**) and 3% Ni (**F**).

FESEM images of CeO_2_ and Ni-doped CeO_2_ nanoparticles are shown in Figure 3. The spherical shape of the synthesized particles can be seen. The FESEM images further indicate that the size of particles was decreased as a function of the increased percentage of Ni doping of the crystal structure. The same conclusion is reached in the XRD discussion.

**Figure 4:**
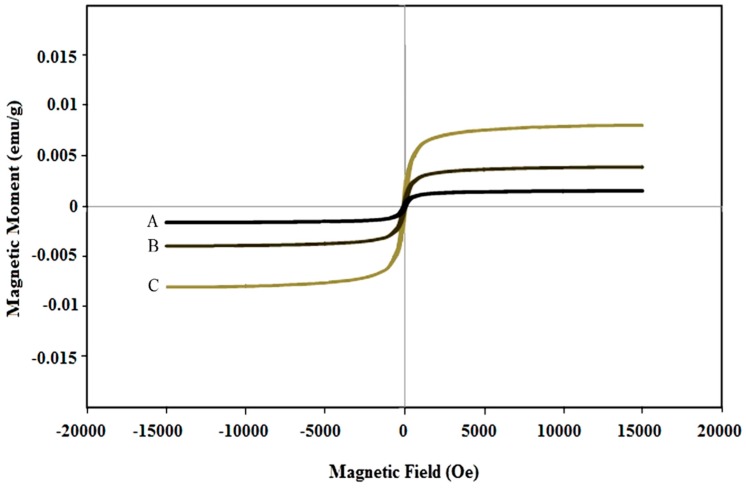
Magnetic hysteresis curves of synthesized nanoparticles of (**A**) CeO_2_ and CeO_2_ doped with 1% Ni (**B**) and 3% Ni (**C**) by using *S. rebaudiana* extract.

Energy-dispersive X-ray spectroscopy (EDX) is an analytical technique used for the elemental analysis or chemical characterization of a sample. This analysis was used to determine the presence of nickel in the structure of cerium oxide nanoparticles. As shown in Figure 4E–H, elemental nickel is clearly observed in the EDX graphs of doped CeO_2_ nanoparticles, which confirms the presence of nickel ion in the CeO_2_ nanoparticles.

The magnetic properties of synthesized nanoparticles of CeO_2_ and Ni-doped CeO_2_ are shown in Figure 4. The magnetic moment (magnetization) as a function of an applied magnetic field exhibited a paramagnetic-type behavior. However, a close inspection of the curves shows that a slight hysteresis exists and that the hysteresis area is proportional to the Ni cation doping level. Thus, the synthesis procedure allows modulating the magnetic features of nanoparticles. The doping effect on the magnetic properties of nanoparticles is enhanced and gradual increase of the weak ferromagnetic behavior is seen. As shown in Figure 4, the small area of the hysteresis loop of the synthesized nanoparticles is further indicative of a weak ferromagnetic material. This behavior can be understood as a function of the double valence of cerium cations, in addition to Ni cation insertion in the crystalline lattice of CeO_2_. In two separate studies, Coey and Sundaresan suggested that increased oxygen vacancies due to the development of reduced cerium Ce^+3^ from Ce^+4^ of lattice can increase the interactions between unpaired spins and thus increase the ferromagnetic behavior of CeO_2_ nanoparticles [36,37].

Sun Protection Factor (SPF) is a standard measurement of the ability of sunscreens to absorb UV rays. By using the spectrophotometric method and Mansur (Equation (1)), one can estimate the value of the SPF parameter for a sample [38]:

SPF = CF × Σ EE(λ) × I(λ) × Abs(λ)(1)

where the correction factor (CF) is 10, EE(λ) is the erythema effect, I(λ) is the intensity of sunlight and abs(λ) is the wavelength absorption. For ease of calculation in this equation, Sayre et al. calculated and presented values of EE(λ) × I(λ) at 290 to 320 nm of wavelength as numbers (Table 1).

**Figure 5:**
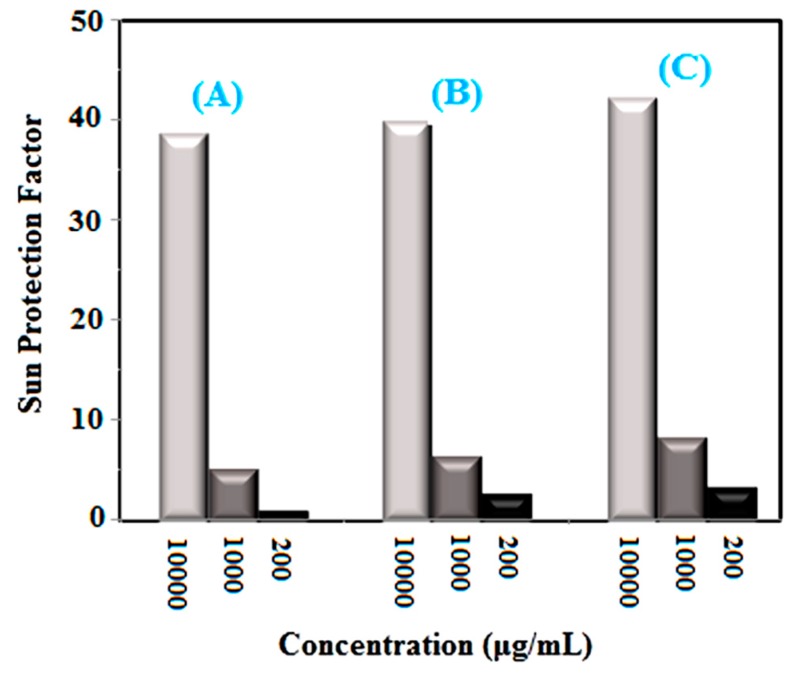
Sun Protection Factor (SPF) of synthesized nanoparticles of (**A**) CeO_2_ and CeO_2_ doped with 1% Ni (**B**) and 3% Ni (**C**) by using *S. rebaudiana* extract.

After calculation at 10,000 μg/mL concentration, the SPF values were derived as being equal to 38.98, 40.15, 42.54 and 43.47 for non-doped, 1, 3 and 5% Ni-doped CeO_2_, respectively (see Figure 5). Mineral filters suitable for sunscreens should exhibit particles with two major features, which include particle size and band-gap. As a matter of fact, a smaller particle size and a larger band-gap are better for a UV absorber. According to previous studies, the band-gap of CeO_2_ nanoparticles is larger than that of titanium oxide and zinc oxide nanoparticles, and the synthesized nanoparticles have 8–10 nm size, so the synthesized nanoparticles can be a better choice for use in sunscreens [39].

## 3. Materials and Methods

### 3.1. Chemicals

The nickel (II) nitrate hexahydrate (Ni(NO_3_)_2_·6H_2_O,) and cerium nitrate hexahydrate Ce(NO_3_)_3_·6H_2_O (50 mL, 99.99%) were prepared from Merck, Darmstadt, Germany.

### 3.2. Nanoparticle Synthesis

**Figure 6:**
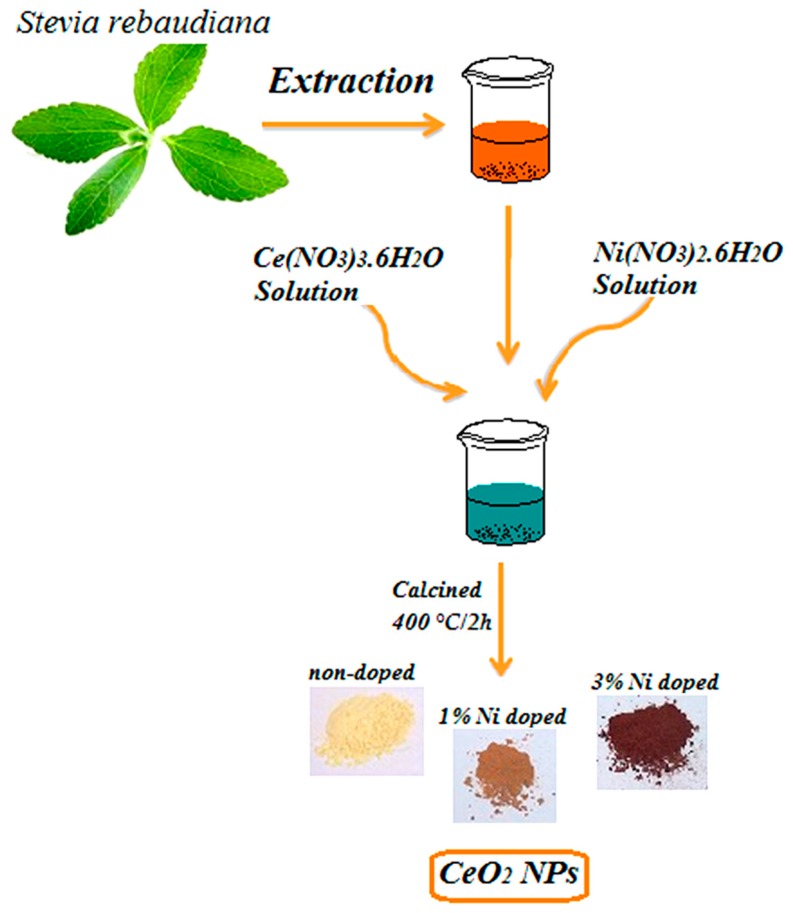
Schematic of the synthesis procedure of CeO_2_ and Ni-doped CeO_2_ nanoparticles by using *S. rebaudiana* extract.

*S. rebaudiana* extract acts as a capping and stabilizing agent for the synthesis of nanoparticles, because of the compounds such as terpenoids, alkaloids, and tannins in its leaves. Hence, cerium species can be stabilized by *S. rebaudiana* extract and form nanoparticles. *S. rebaudiana* leaves were collected, dried and crushed. Powder plant was soaked in distilled water (1:10 ratio). Then, the mixture was centrifuged at 150 rpm for 24 h and filtered via Whatman paper. In the nanoparticle synthesis, *Stevia* extract (10 mL) was diluted with distilled water (50 mL), and 0.1 M cerium nitrate hexahydrate Ce(NO_3_)_3_·6H_2_O (50 mL) was added to the solution. Then 0, 1, 3 and 5% *w*/*w* of Ni to CeO_2_ was synthesised adding nickel (II) nitrate hexahydrate (Ni(NO_3_)_2_·6H_2_O) to the same solution, using individual baths. The solutions were heated up to 80 °C for 5 h. In the last step, the mixture was dried at 90 °C giving the precursor powders, which were calcined at 400 °C during 2 h, in an air atmosphere, using a conventional furnace. A schematic diagram of the major steps of synthesis is show in Figure 6. Nanoparticles of CeO_2_ and Ni doped CeO_2_ was synthesized by using ***S. rebaudiana*** extract.

### 3.3. Characterization

The synthesized nanoparticles were characterized through X-ray diffraction on an X’Pert PRO MPD system (PANalytical, Almelo, The Netherlands), by Scanning Electron Microscopy (SEM) using a model MIRA3 instrument (Tescan, Czech Republic), a Raman model Takram P50C0R10 (Teksan, Tehran, Iran) at 532 nm leaser wavelength, a model Tensor27 FT-IR instrument (Bruker, Optics GmbH, Ettlingen, Germany) and a model 1800 UV-Vis spectrometer (Shimadzu, Kyoto, Japan).

### 3.4. Ultraviolet Protection Level of CeO_2_ and Ni Doped CeO_2_ Nanoparticles

The survey of UV protection of nanoparticles was done by using Sun Protection Factor (SPF) determination *in vitro*. For purpose the synthesized sample (1 g) was dissolved using ethanol by ultrasonic irradiation for 15 min, then the prepared solution (5 mL) diluted using ethanol (50 mL). In following, this solution (5 mL) was diluted using 25 mL of ethanol. Absorption values were measured through spectrophotometry in the 290 to 320 nm range [31].

## 4. Conclusions

Nanometric particles of CeO_2_ and Ni-doped CeO_2_ solid solutions were synthesized via a green chemistry route using *S. rebaudiana* extract. Doping of CeO_2_ nanoparticles leads to the development of ferromagnetic properties. Also, the UV protection property of the CeO_2_ nanoparticles improved and increased by doping Ni into the fluorite structure of these nanoparticles, making Ni-doped CeO_2_ nanoparticles a suitable choice for use in sunscreens.

## Figures and Tables

**Table 1:** List of the normalized multiplication function values used in calculating the Sun Protection Factor (SPF).

Wavelength (nm)	290	295	300	305	310	315	320
EE × I (Normalized)	0.0150	0.0817	0.2874	0.3278	0.1864	0.0837	0.0180
